# S6 ribosomal protein phosphorylation is associated with malignancy of intraductal papillary mucinous neoplasm of the pancreas

**DOI:** 10.1002/ags3.12367

**Published:** 2020-06-24

**Authors:** Teijiro Hirashita, Yuka Hirashita, Yukio Iwashita, Yuichi Endo, Maki Kiyonaga, Shunro Matsumoto, Naoki Hijiya, Masatsugu Moriyama, Kazunari Murakami, Masafumi Inomata

**Affiliations:** ^1^ Department of Gastroenterological and Pediatric Surgery Faculty of Medicine Oita University Yufu Japan; ^2^ Gastroenterology Faculty of Medicine Oita University Yufu Japan; ^3^ Radiology Faculty of Medicine Oita University Yufu Japan; ^4^ Molecular Pathology Faculty of Medicine Oita University Yufu Japan

**Keywords:** 18F‐Ffluorodeoxyglucose positron emission tomography, glucose transporter 1, intraductal papillary mucinous neoplasms, mammalian target of rapamycin complex 1, phosphorylated S6 ribosomal protein

## Abstract

**Background:**

Glucose metabolism of intraductal papillary mucinous neoplasms (IPMNs) of the pancreas is unclear. S6 ribosomal protein (S6) phosphorylation is involved not only in controlling cell growth but also in glucose metabolism in cancer. The aim of this study was to investigate the role of S6 phosphorylation and the significance of glucose metabolic changes in IPMN.

**Methods:**

Records of 39 patients who underwent preoperative FDG‐PET and curative resection were enrolled in this study. S6 phosphorylation and GLUT1 expression were evaluated immunohistochemically in these patients. The effect of S6 phosphorylation on glucose uptake was examined in cancer cell lines. To examine the change of glucose metabolism in IPMN clinically, the relation between clinical factors including FDG‐PET and malignancy of IPMN was investigated.

**Results:**

S6 phosphorylation and GLUT1 expression were significantly higher in carcinoma than in normal cells or adenoma. Cell lines with high level of S6 phosphorylation showed high glucose uptake, and inhibition of S6 phosphorylation reduced glucose uptake. In clinical examination, FDG‐PET was the independent factor related to the diagnosis of adenoma or carcinoma (odds ratio = 20.0, 95% confidence interval = 1.837‐539.9, *P* = .012). FDG‐PET detected carcinoma with a sensitivity of 81.8%, specificity of 96.4%, and accuracy of 92.3%.

**Conclusion:**

S6 phosphorylation was associated with glucose uptake and malignancy of IPMN. Moreover, glucose uptake increased in malignant cells of IPMN, and FDG‐PET is useful for detecting malignancy of IPMN.

## Graphical abstract

The role of S6 phosphorylation in IPMN and the relation between glucose metabolism and malignancy of IPMN is unclear. We showed S6 phosphorylation was associated with glucose uptake and malignancy of IPMN, and FDG‐PET is useful for detecting malignancy of IPMN.

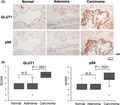

## 1 INTRODUCTION

Intraductal papillary mucinous neoplasms (IPMNs), which are commonly identified cystic pancreatic neoplasms, are precursors to invasive carcinoma.^1^, ^2^ IPMNs are characterized by mucin‐producing dysplastic epithelium originating from the main pancreatic duct or branched pancreatic duct.^3^ Although the management of IPMNs has been maintained over recent years, revisions of international consensus guidelines for the management of IPMN of the pancreas^4^ have defined worrisome clinical and radiological features and high‐risk stigmata of malignancy that aid in clinical decision making. However, some patients with IPMN do not fall within this diagnostic algorithm, and we sometimes need more information on whether the IPMN is malignant.

Cellular metabolism is one of the main processes that is affected during the transition from normal to cancer cells. In particular, glucose metabolism is often altered in tumor cells, and thus, glucose uptake increases. Although the mechanism of the cancer cells' glucose dependency remains unknown, many reports have indicated that glucose uptake is associated with glycolytic enzymes including glucose transporter 1 (GLUT1) in various cancer cells.^5^ Phosphorylated S6 ribosomal protein (pS6) indicates mechanistic target of rapamycin complex 1 (mTORC1) activity, and mTORC1 plays a central role in controlling cells growth. Additionally, recent studies have shown that the mTORC1 is a key regulator of glucose metabolism in cancer.^6^, ^7^, ^8^

18F‐Fluorodeoxyglucose positron emission tomography (FDG‐PET) can detect increases of glucose uptake, and therefore, it should be useful in detecting tumor tissue. Some reports have shown the usefulness of FDG‐PET in the diagnosis of the malignancy of IPMN,^5^, ^9^ but the role of S6 phosphorylation in IPMN and the relation between glucose metabolism and malignancy of IPMN is unclear. The aim of this study was to clarify the effect of S6 phosphorylation on glucose metabolism and malignancy of IPMN, and to assess the significance of glucose metabolic changes in IPMN clinically.

## 2 PATIENTS AND METHODS

### 2.1 Patients

In total, 39 patients with IPMN who underwent preoperative FDG‐PET and curative resection in the Department of Gastroenterological and Pediatric Surgery, Oita University were enrolled in this study. Clinicopathological features including patient characteristics, radiological findings, operative procedures, and pathological findings were examined retrospectively. We followed the ethical principles stated in the guidelines of the World Medical Association's Declaration of Helsinki in this study.

### 2.2 Immunohistochemistry

Immunohistochemistry was performed using rabbit polyclonal antibodies against GLUT1 diluted 1:100 (Abcam, Tokyo, Japan) and pS6 (S240/S244) diluted 1:400 (Cell Signaling Technology, Danvers, MA, USA). Briefly, after antigen retrieval and inactivation of endogenous peroxidase, tissue sections were blocked with 2.5% horse serum (ImmPRESS Reagent Kit; Vector Laboratories Inc, Burlingame, CA, USA) at room temperature (RT) for 30 minutes and then incubated at 4°C overnight with antibodies against GLUT1 and pS6 diluted in SignalStain Antibody Diluent (Cell Signaling Technology) and 1% bovine serum albumin/PBS, respectively. After washing in PBS, the sections were incubated at RT for 30 minutes with peroxidase‐conjugated horse anti‐rabbit Ig (ImmPRESS Reagent Kit; Vector Laboratories Inc). Peroxidase activity was detected using ImmPACT DAB Peroxidase Substrate (Vector Laboratories Inc).

### 2.3 Evaluation of immunohistochemistry

The slides were reviewed by two independent observers who had no knowledge of patient outcomes. Expression of GLUT1 and phosphorylation of S6 were observed and scored in normal epithelial cells, adenoma, and carcinoma in the patients with IPMNs. The staining intensity was scored as: 0, no staining; 1, weak (less than that in fibroblasts in the positive internal control); 2, moderate (as intense as that in the positive internal control cells); or 3, strong (more than that in the positive internal control cells). The staining population was scored according to the ratio of positive cells as follows: 0, 0% of cells positive; 1, 1%–9% of cells positive; 2, 10%–50% of cells positive; and 3, >50% of cells positive. Scoring was carried out in three distinct fields per case, and the three scores were averaged and rounded off to the nearest whole number. The sums of the scores for intensity and population were defined as the GLUT1 and pS6 scores in this study.

### 2.4 Cell lines

The human pancreatic cancer cell line BxPC‐3 was obtained from the ATCC. PK‐45H and PANC‐1 cells were obtained from RIKEN BRC through the National Bio‐Resource Project of MEXT, Japan. SUIT‐2, TCC‐Pan2, and KP‐3L cells were obtained from the Japanese Collection of Research Bioresources. All cell lines were cultured under conditions recommended by the providers.

### 2.5 Glucose uptake assay

Cells were seeded at densities of 2 × 10^4^ cells/well in 96‐well plates. They were incubated for 24 hours and then the medium was replaced with glucose‐free RPMI (with DMSO or PF04691502 [1 µM, Selleck Chemicals, Houston, TX, USA]) for 6 hours. PF04691502 treatment decreases in mTORC1 activity, which means inhibition of S6 phosphorylation. The amount of glucose uptake in the different cell lines was measured using a 2‐NBDG Glucose Uptake Assay Kit (Bio Vision, California, USA).

### 2.6 Western blotting

Western blotting was performed as described previously.^10^ Cells were lysed on ice for 20 minutes in SDS‐modified RIPA buffer containing protease and phosphatase inhibitor cocktails (cOmplete Mini and PhosSTOP EASYpack, respectively; Roche Diagnostics, Mannheim, Germany) and then centrifuged at 12,000 *g* at 4°C for 20 minutes. The resulting cell lysates (20 µg samples) were boiled with Laemmli sample buffer and subjected to SDS‐PAGE. The samples were transferred to a polyvinylidene difluoride membrane (Millipore), which was blocked for 1 hour in Block Ace (DS Pharma, Osaka, Japan) at RT, and then incubated overnight at 4°C with primary antibodies against pS6 (1:2000, S235/S236), S6 (1:1000, Cell Signaling Technology), and GAPDH (1:1000, Santa Cruz Biotechnology, Santa Cruz, CA, USA). After washing with 1 × TBS containing 0.1% Tween 20, membranes were incubated for 1 hour at RT with appropriate secondary antibodies, followed by rewashing. Finally, the signals were detected using an ECL Western blotting analysis system (GE Healthcare, Piscataway, NJ, USA) in accordance with the manufacturer's instructions.

### 2.7 Cell proliferation assay

Cell proliferation assay were performed as described previously.^10^ Cells were incubated with DMSO or the serially diluted PF04691502 (0.1, 1.0, and 10 μmol/L).

### 2.8 Statistical analysis

We investigated the relationship between the clinical factors and diagnosis (benign or malignant) of IPMN using univariate and multivariate analysis. IPMN was classified as intraductal papillary mucinous adenoma (IPMA) and intraductal papillary mucinous adenocarcinoma (IPMC) according to Classification of Pancreatic Carcinoma 4th English Edition.^11^ IPMA included IPMN with low‐ or intermediate‐grade dysplasia, and IPMC included IPMN with high‐grade dysplasia and an associated invasive carcinoma.^12^

We included the following 18 perioperative factors in the analyses: age, sex, body mass index (BMI), serum levels of CEA and CA19‐9, HbA1c, symptoms except jaundice, jaundice, type of IPMN (main duct [MD‐IPMN]/branch duct [BD‐IPMN]/mixed), location of tumor (head/body and/or tail/whole), diameter of main pancreatic duct, diameter of cyst, enhanced nodule, thickness of the cystic wall, dilatation of the common bile duct, maximum standardized uptake value (SUV‐max) of FDG‐PET, and surgical procedure (pancreatoduodenectomy (PD)/distal pancreatectomy (DP)/others).

The sensitivity, specificity, and accuracy were calculated for FDG‐PET. The cutoff point for SUV‐max was determined using receiver operating characteristic (ROC) curve analysis.

All variables are expressed as mean ± standard deviations for continuous data. Prior to analysis, continuous data were divided into two groups using averages or abnormal values. The correlation among the continuous variables was investigated using the Spearman's rank correlation coefficient. Univariate analyses were performed using the Student’s *t*‐test or the Tukey test for continuous variables and chi‐squared test for categorical variables. The results of the multivariate logistic regression analysis are expressed as adjusted odds ratios with 95% confidence intervals. Survival analysis was performed using Kaplan‐Meier estimated survival. Statistical significance was defined as *P* < .05. All statistical analyses were performed using JMP^®^ 11 (SAS Institute Inc, Cary, NC, USA).

## 3 RESULTS

### 3.1 Clinicopathological features

Mean patient age was 71.5 ± 9.0 years. Of the 39 patients, 18 were women and 21 were men (Table 1). Serum levels of CEA and CA19‐9 were 4.1 ± 4.5 ng/mL and 138.3 ± 516.8 U/mL, respectively. Four patients had jaundice (10%). Two patients were classified as having MD‐IPMN, 18 (46%) as having BD‐IPMN, and 19 (49%) as mixed. Tumors were located at the head of the pancreas in 21 patients (54%), body and/or tail in 15 (38%), and whole pancreas in three patients (8%). The mean diameters of the main pancreatic ducts and cysts were 8.0 ± 6.2 mm and 26.8 ± 17.9 mm, respectively. Enhanced nodules were recognized in 34 patients (87%). The mean SUV‐max of FDG‐PET was 6.9 ± 5.8. PD was performed in 23 patients (59%), DP in 14 (36%), and other procedures in two patients (5%). Ten patients (23%) were diagnosed pathologically as having intraductal papillary mucinous adenoma (IPMA) and 29 (74%) as having intraductal papillary mucinous adenocarcinoma (IPMC).

**TABLE 1:** Patient characteristics (n = 39)

Characteristic	Value
Age (y)	71.5 ± 9.0
Sex (female/male)	18 (46%)/21 (54%)
Body mass index (kg/m^2^)	23.5 ± 3.2
CEA (ng/mL)	4.1 ± 4.5
CA19‐9 (U/mL)	138.3 ± 516.8
HbA1c (%)	6.4 ± 1.0
Symptom (−/+)	25 (64%)/14 (36%)
Jaundice (−/+)	35 (90%)/4 (10%)
Type of IPMN (MD/BD/mix)	2 (5%)/18 (46%)/19 (49%)
Location of tumor (head/body, tail/whole)	21 (54%)/15 (38%)/3 (8%)
Diameter of main pancreatic duct (mm)	8.0 ± 6.2
Diameter of cyst (mm)	26.8 ± 17.9
Enhanced nodule (−/+)	5 (13%)/34 (87%)
Thickness of cystic wall (−/+)	22 (56%)/17 (44%)
Dilatation of common bile duct (−/+)	34 (87%)/5 (13%)
PET/CT (SUV‐max)	6.9 ± 5.8
Operation (PD/DP/others)	23 (59%)/14 (36%)/2 (5%)
Diagnosis (IPMA/IPMC)	10 (26%)/29 (74%)

Abbreviations: IPMN, intraductal papillary mucinous neoplasm; MD, main duct; BD, branch duct; PET/CT, positron emission tomography with computed tomography; SUV‐max, maximum standardized uptake value; PD, pancreatoduodenectomy; DP, distal pancreatectomy; IPMA, intraductal papillary mucinous adenoma; IPMC, intraductal papillary mucinous adenocarcinoma.

### 3.2 Immunohistochemical evaluation of GLUT1 and pS6 in IPMN

**FIGURE 1:**
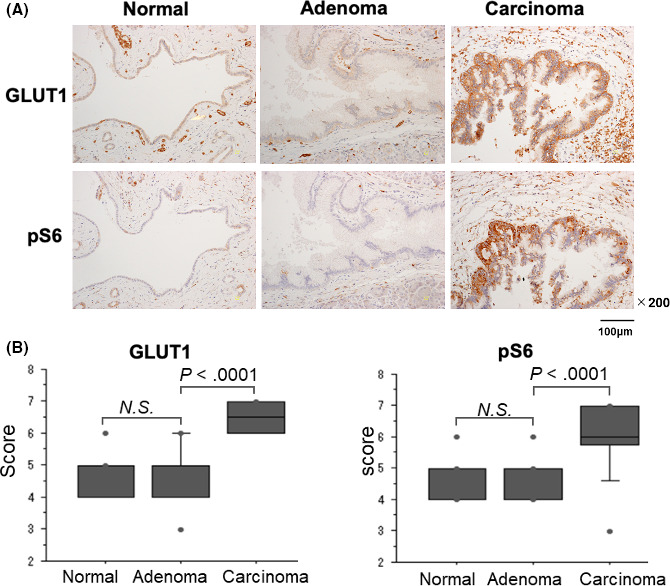
Immunohistochemistry for GLUT1 and pS6. (A) Immunohistochemical staining for GLUT1 and pS6 in normal cells, adenoma, and carcinoma. (B) The level of GLUT1 expression and S6 phosphorylation were significantly higher in carcinoma than in normal cells or adenoma

Immunohistochemical results of the level of GLUT1 and pS6 are shown in Figure 1A, B. There were no significant differences in the expression of GLUT1 between normal epithelium and adenoma (*P* = .1382), and its expression was significantly higher in carcinoma than in normal epithelium (*P* < .001). The level of pS6 was also increased in carcinoma compared to adenoma (*P* < .001).

### 3.3 Relation between S6 phosphorylation and glucose uptake and cell in pancreatic cancer cell lines

**FIGURE 2:**
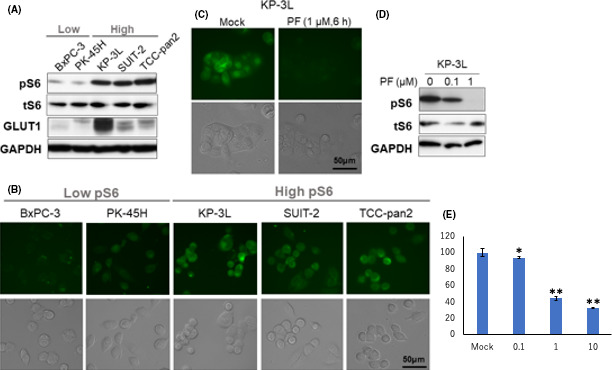
(A) Western blotting for the examination of GLUT1 expression and S6 phosphorylation in 5 pancreatic cancer cell lines. Three of the 5 cell lines showed high levels of GLUT1 expression and S6 phosphorylation. (B) Glucose uptake assay for the cell lines. The 3 cell lines with high expression of GLUT1 and S6 phosphorylation showed higher glucose uptake than the other 2 cell lines. (C, D) Glucose uptake assay and Western blotting for examination of the effect of S6 phosphorylation on glucose uptake using PF04691502 treatment. Glucose uptake decreased in KP‐3L cells treated with PF04691502. (E) Inhibitory effect of PF04691502 treatment on cell proliferation in KP‐3L cells. Cell proliferation was significantly reduced. *vs control,* P* < .05, ** vs control,* P* < .001

GLUT1 expression and S6 phosphorylation were examined in five pancreatic cancer cell lines by Western blotting. Three of the five cell lines showed high levels of GLUT1 expression. The level of S6 phosphorylation was also high in these three cell lines with high GLUT1 expression (Figure 2A). Higher levels of glucose uptake were observed in these three cell lines than in the other two cell lines, which showed low level of S6 phosphorylation (Figure 2B). We also investigated the effect of inhibition of S6 phosphorylation on glucose uptake using PF04691502 treatment. Glucose uptake in KP‐3L, which showed high levels of S6 phosphorylation, was markedly reduced after treatment with PF04691502 (Figure 2C, D). KP‐3L was effectively reduced by the PD0325901 treatment in cell proliferation assay (Figure 2E). The same examination was conducted for TCC‐Pan 2, and glucose uptake and cell proliferation reduced after treatment with PF04691502 (Figure S1).

### 3.4 Correlation between the GLUT1, pS6, and SUV‐max of FDG‐PET

**FIGURE 3:**
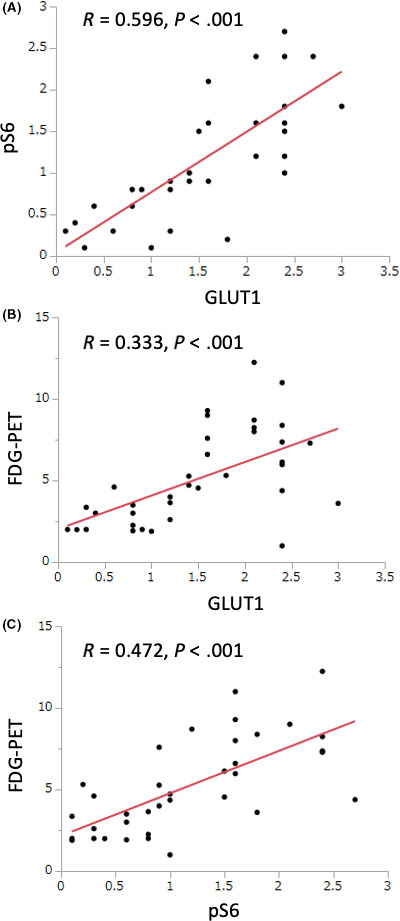
A scatter plot showing the relationship between GLUT1 and pS6, GLUT1 and SUV‐max of FDG‐PET, and pS6 and SUV‐max of FDG‐PET

Spearman's rank correlations between the GLUT1, pS6, and SUV‐max of FDG‐PET are shown in Figure 3. Significant correlations were found between GLUT1 and pS6 (*R *= 0.596, *P* < .001), GLUT1 and SUV‐max of FDG‐PET (*R* = 0.333, *P* < .001), and pS6 and SUV‐max of FDG‐PET (*R* = 0.472, *P* < .001), respectively.

### 3.5 Relation between SUV‐max of FDG‐PET and diagnosis of IPMN

In the analysis of SUV‐max of FDG‐PET, the cutoff point was 3.5 as determined by ROC curve analysis. FDG‐PET detected IPMC with a sensitivity of 81.8%, specificity of 96.4%, and accuracy of 92.3% (Table 2).

**TABLE 2:** Relation between PET/CT and IPMN

	SUV‐max < 3.5	SUV‐max ≥ 3.5
IPMA	9	1
IPMC	2	27
	Sensitivity 81.8%	Specificity 96.4%

Abbreviations: PET/CT, positron emission tomography with computed tomography; IPMN, intraductal papillary mucinous neoplasm; IPMA, intraductal papillary mucinous adenoma; IPMC, intraductal papillary mucinous adenocarcinoma.

### 3.6 Relation between clinical factors and diagnosis of IPMN

Univariate analyses showed that enhanced nodule, thickness of the cystic wall, and SUV‐max of PET/CT were significant factors related to the diagnosis of IPMA or IPMC (Table 3). Multivariate analysis revealed that SUV‐max of PET/CT was the sole independent factor related to the diagnosis of IPMA or IPMC (odds ratio = 20.0, 95% confidence interval = 1.837‐539.9, *P* = .012).

**TABLE 3:** Univariate and multivariate analyses for diagnosis of IPMN

Variable	Univariate			Multivariate	
	IPMA (n = 10)	IPMC (n = 29)	*P* value	OR (95% CI)	*P* value
Age (y)					
<70 y	6 (60%)	11 (38%)	.226		
≥70 y	4 (40%)	18 (62%)			
Sex (female/male)					
Female	3 (30%)	14 (48%)	.229		
Male	7 (70%)	15 (52%)			
Body mass index					
<25 kg/m^2^	7 (70%)	22 (76%)	.717		
≥25 kg/m^2^	3 (30%)	7 (24%)			
CEA (ng/mL)					
<5.0 ng/mL	9 (90%)	23 (79%)	.425		
≥5.0 ng/mL	1 (10%)	6 (21%)			
CA19‐9					
<37 U/mL	9 (90%)	19 (66%)	.111		
≥37 U/mL	1 (10%)	10 (34%)			
HbA1c					
<6.1%	4 (40%)	13 (45%)	.790		
≥6.1%	6 (60%)	16 (55%)			
Symptom					
Yes	2 (20%)	12 (41%)	.209		
No	8 (80%)	17 (59%)			
Jaundice					
Yes	0 (0%)	4 (14%)	.112		
No	10 (100%)	25 (86%)			
Type of IPMN					
MD	2 (20%)	0 (0%)	.056		
BD	4 (40%)	14 (48%)			
Mix	4 (40%)	15 (52%)			
Location of tumor					
Head	4 (40%)	17 (59%)	.546		
Body and/or tail	4 (40%)	11 (38%)			
Whole	2 (20%)	1 (3%)			
Diameter of main pancreatic duct					
<10 mm	6 (60%)	22(76%)	.347		
≥10 mm	4 (40%)	9 (24%)			
Diameter of cyst					
<30 mm	7 (70%)	18 (62%)	.649		
≥30 mm	3 (30%)	11 (38%)			
Enhanced nodule					
Yes	5 (50%)	29 (100%)	<.0001	0.000 (0.000‐1.677)	.095
No	5 (50%)	0 (0%)			
Thickness of cystic wall					
Yes	0 (0%)	17 (59%)	0.0002	0.000 (0.000‐3.663)	0.165
No	10 (100%)	12 (41%)			
Dilatation of common bile duct					
Yes	0 (0%)	5 (13%)	.073		
No	10 (100%)	24 (83%)			
PET/CT (SUV‐max)					
<3.5	9 (90%)	2 (7%)	<.0001	20.00 (1.837‐539.9)	.012
≥3.5	1 (10%)	9 (93%)			
Operation					
PD	6 (60%)	17 (59%)	.536		
DP	4 (40%)	10 (34%)			
Others	0 (0%)	2 (7%)			

Abbreviations: IPMN, intraductal papillary mucinous neoplasm; IPMA, intraductal papillary mucinous adenoma; IPMC, intraductal papillary mucinous adenocarcinoma; OR, odds ratio; CI, confidence interval; MD, main duct; BD, branch duct; PET/CT, positron emission tomography with computed tomography; SUV‐max, maximum standardized uptake value; PD, pancreatoduodenectomy; DP, distal pancreatectomy.

### 3.7 Relations between FDG‐PET, GLUT1, and pS6 and prognosis

**FIGURE 4:**
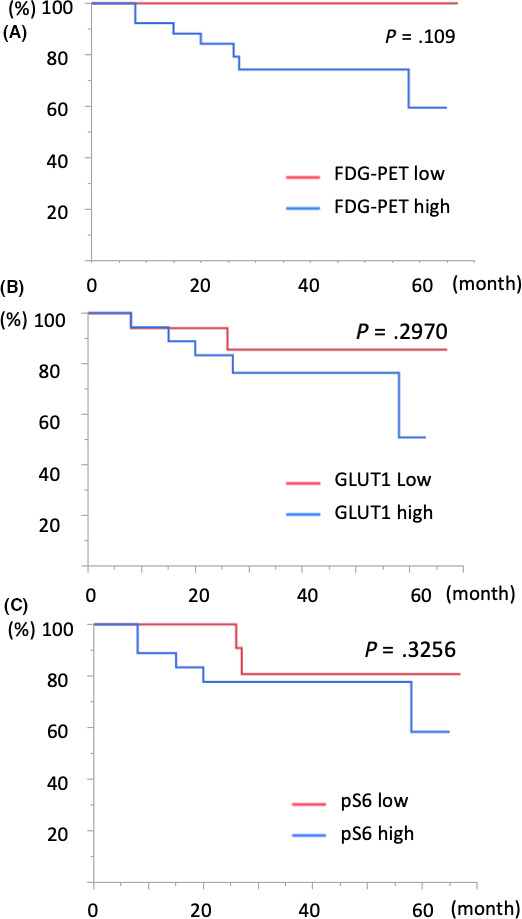
Relations between FDG‐PET, GLUT1, and pS6 and prognosis. The rates of overall survival were not significantly different between high and low levels of FDG‐PET, GLUT1, and pS6, respectively

GLUT1 expression and S6 phosphorylation were divided into two groups using median values. Regarding the patients with IPMN, the rates of overall survival were not significantly different between high and low levels of FDG‐PET (*P* = .109), GLUT1 (*P* = .297), and pS6 (*P* = .326), respectively (Figure 4). Even in patients with IPMC alone, the rates of overall survival were not significantly different between high and low levels of these three factors (Figure S2).

## 4 DISCUSSION

As indications for the resection of IPMN, the Fukuoka consensus criteria are useful for predicting malignancy.^1^, ^4^ IPMN with high‐risk stigmata, such as enhanced mural nodules (≥5 mm), dilation of the main pancreatic duct (≥10 mm), or obstructive jaundice in a patient with a cystic lesion at the head of pancreas, should be resected in surgically fit patients. IPMN with worrisome features, such as a large cyst (≥30 mm), enhanced mural nodule (<5 mm), thickened enhanced cystic wall, dilation of main pancreatic duct (5‐9 mm), abrupt change in the caliber of the main pancreatic duct with distal pancreatic atrophy, lymphadenopathy, an elevated serum level of CA19‐9, and rapid rate of cystic growth (>5 mm/2 years), should be evaluated and closely observed. These factors usually can be revealed using enhanced computed tomography (CT) or endoscopic ultrasonography. However, we sometimes encountered patients in the past whose treatment strategies were difficult to determine. We were especially worried about treatment strategies for elderly patients or patients with comorbidities because few of these patients with high‐risk stigmata were diagnosed as IPMA after resection. Therefore, other modalities are sometimes needed to diagnose malignancies, and some reports showed that other factors, such as a diagnosis of diabetes mellitus and 18F‐FDG accumulation on FDG‐PET, are useful for discriminating between IPMA and IPMC.^13^, ^14^

In this series of patients with IPMN, high SUV‐max of FDG‐PET was significantly associated with the malignancy of IPMN, which was independent of known worrisome features or high‐risk stigmata. The link between SUV‐max and carcinogenesis is related to differences in glucose metabolism in cancer cells.^15^ FDG‐PET is useful for detecting various malignancies, and one report showed the usefulness of FDG‐PET in the diagnosis of IPMN.^16^ FDG‐PET detected IPMC with a sensitivity of 61.5%–90.0%, and specificity of 77.8%–100% in recent reports.^8^, ^16^, ^17^, ^18^, ^19^ Our data showed that FDG‐PET detected IPMC with a sensitivity of 85.7% and specificity of 95.8%. These results indicate that FDG‐PET could be useful for detecting malignancies in IPMNs. Although FDG‐PET has some disadvantages in terms of its costs and exposure to radiation, it is a noninvasive modality that can be used to detect not only the local tumor but also metastases.

In this study, we found the expression of GLUT1 and S6 phosphorylation to be upregulated during the adenoma‐carcinoma sequence in IPMN of the pancreas. Moreover, significant correlations between GLUT1 expression, S6 phosphorylation, and SUV‐max of FDG‐PET were shown. These three factors had no relationship with prognosis in patients with IPMN. Significant correlation between FDG uptake and GLUT1 expression was reported in a previous study,^5^ and GLUT1 was reported to correlate with biological behavior in various malignancies.^20^, ^21^ An increase in GLUT1 expression has proved to be associated with tumor aggressiveness and poor prognosis in various carcinomas such as those of the pancreas, lung, and colorectum.^22^, ^23^, ^24^, ^25^

This study also showed an increase in the level of pS6 in carcinoma compared to normal cells or adenoma of IPMN. S6 is a downstream molecule of the PI3K/Akt/mTOR pathway, and phosphorylation GLUT1 expression and S6 phosphorylation were examined in five pancreatic cancer cell lines by Western blotting. Three of the five cell lines showed high levels of GLUT1 expression. The level of S6 phosphorylation was also high in these three cell lines with high GLUT1 expression (Figure 2A). Higher levels of glucose uptake were observed in these three cell lines than in the other two cell lines, which showed low level of S6 phosphorylation (Figure 2B). We also investigated the effect of the decrease in mTORC1 activity on glucose uptake using PF04691502 treatment, which inhibits PI3K/mTOR. Glucose uptake in KP‐3L and TCC pan 2, which showed high levels of mTORC1 activity, was markedly reduced after treatment with PF04691502 (Figure 2C, D).

S6 indicates the activation of mTORC1. mTORC1 activity is known as the key regulator of glucose metabolism, and it correlates with glucose addiction.^26^ We showed in vitro that an inhibition of S6 phosphorylation by PF04691502 treatment reduced glucose uptake. These results supported the notion that not only the expression of GLUT1 but also mTORC1 activity contribute to glucose uptake in carcinoma cells of IPMN. Recently, mutations in KRAS and GNAS have been identified in the tissue and cyst fluid of patients with IPMN. These alterations are known to induce activation of the PI3K/AKT/mTOR signaling pathway.^27^ Therefore, in IPMN, activation of mTORC1 may also have a potential pivotal role in the carcinogenesis of IPMN. Additional study of the mechanism of upregulation of mTORC1 activity in IPMN will be needed.

Previous studies have examined whether inhibition of glucose uptake is useful in treating cancer.^28^ Glucose uptake through the plasma membrane plays a key role for glucose consumption by cancer cells; therefore, GLUT1 has the potential to be an ideal point for targeted therapy. Recent studies showed that inhibition of GLUT1 decreased cancer cells, such as pancreatic cancer, lung cancer, and ovarian cancer; however, these effects have only been observed in vivo.^28^, ^29^ Regarding the therapeutic effect of PF04691502 through inhibition PI3K/mTOR, clinical trials for some carcinomas were reported.^30^, ^31^ However, the efficacy has not been proven and a high rate of adverse events has been reported. There is a possibility that it can be used by consideration of administration method or combination therapy with the other anti‐cancer agents. Further study will be needed for treatment with inhibition of glucose uptake.

Currently, FDG‐PET can be performed clinically. We evaluated clinical factors including FDG‐PET using multivariate analysis without S6 phosphorylation and GLUT1 expression to show the usefulness of detecting glucose uptake. S6 phosphorylation and GLUT1 expression have a key role of glucose uptake; therefore, they could be a biomarker of malignancy of IPMN when S6 phosphorylation or GLUT1 expression can be easily detected using pancreatic juice in the future.

This study has several limitations. Sample size was small and all types of IPMN were analyzed simultaneously. The incidence of patients with MD‐IPMN was less compared with BD‐IPMN and mixed type IPMN in this study. Further study will be needed to clarify the role of S6 phosphorylation or FGD‐PET depending on the type of IPMN.

In conclusion, S6 phosphorylation was associated with glucose metabolism and malignant potential of IPMN. Moreover, glucose uptake increased in malignant cells of IPMN, and FDG‐PET is useful for detecting malignancy in IPMN.

## DISCLOSURE

Conflict of interest: The authors declare no conflict of interests related to this article.

## ETHICAL APPROVAL

This study was approved by the ethics committee of Oita University Faculty of Medicine (approval number: 1501).

## Supporting information

Fig S1

Fig S2
