# Supplementary figures and images for: S6 ribosomal protein phosphorylation is associated with malignancy of intraductal papillary mucinous neoplasm of the pancreas
Source: Ann Gastroenterol Surg. 2020 Jun 24;4(5):571–9. doi: 10.1002/ags3.12367 (PMC7511561; doi:10.1002/ags3.12367)

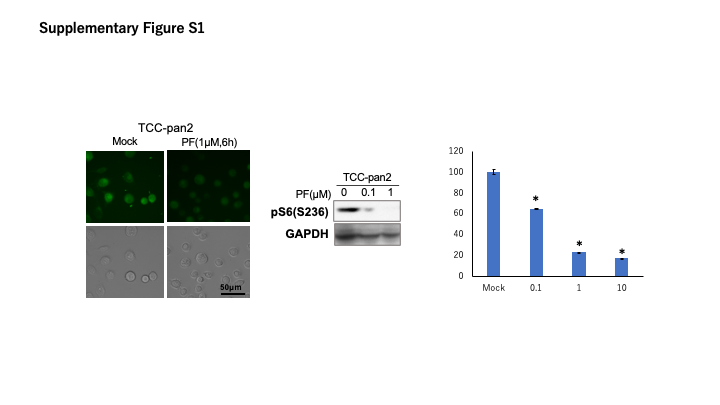

Supplement: Supplementary file 1 — Fig S1 [file AGS3-4-571-s001.tiff]

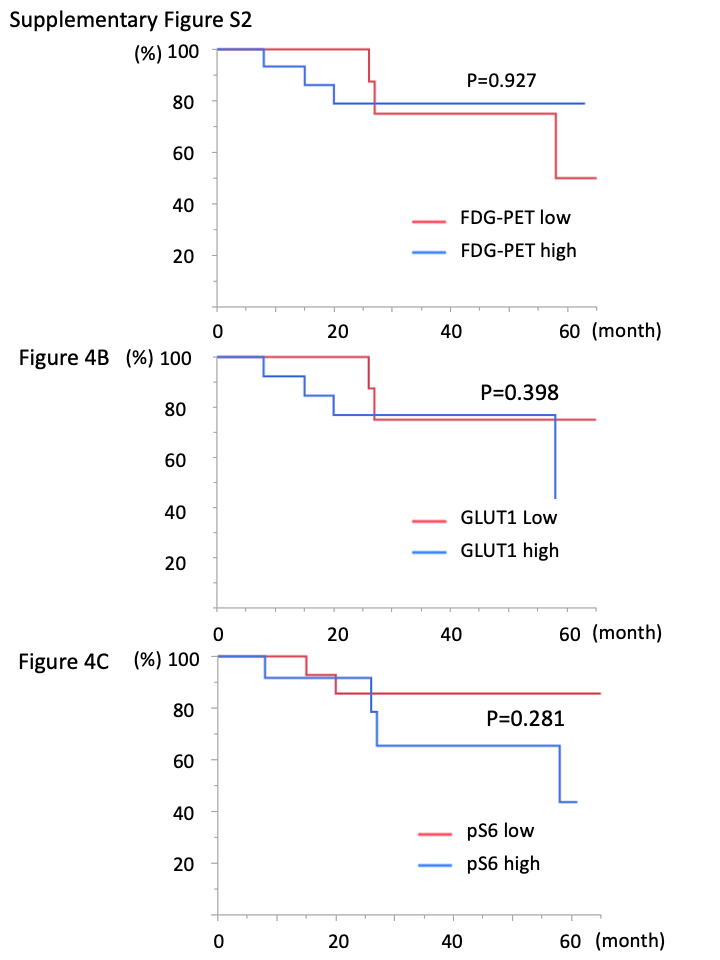

Supplement: Supplementary file 2 — Fig S2 [file AGS3-4-571-s002.tiff]
